# Examination of diaphragm thickness, mobility and thickening fraction in individuals with COPD of different severity

**DOI:** 10.55730/1300-0144.5435

**Published:** 2022-05-22

**Authors:** Ceyhun TOPCUOĞLU, Eylem TÜTÜN YÜMİN, Mustafa HİZAL, Suat KONUK

**Affiliations:** 1 Department of Physiotherapy and Rehabilitation, Faculty of Health Sciences, Munzur University, Tunceli, Turkey; 2 Department of Physiotherapy and Rehabilitation, Faculty of Health Sciences, Bolu Abant İzzet Baysal University, Bolu, Turkey; 3 Department of Radiology, Faculty of Medicine, Bolu Abant İzzet Baysal University, Bolu, Turkey; 4 Department of Chest Diseases, Faculty of Medicine, Bolu Abant İzzet Baysal University, Bolu, Turkey

**Keywords:** Diaphragm thickness, diaphragm mobility, diaphragm thickening fraction, COPD, respiratory muscle function

## Abstract

**Background/aim:**

Diaphragm thickness and mobility assessed by ultrasound in individuals with Chronic Obstructive Pulmonary Disease (COPD) reflect the function of the diaphragm. The aim of this study is to compare the diaphragm thickness, mobility, and thickening fraction in individuals with COPD of different severity and healthy individuals and examine the relationship between these parameters and pulmonary function test parameters.

**Materials and methods:**

A cross-sectional observational study design was used. Thirty individuals (mild = 11; moderate = 13; severe = 6) with COPD and 29 healthy male individuals aged between 40–75 years were included in the study. The individuals included in the study were evaluated between October 2020/May 2021. Pulmonary functions were measured with a spirometer, while diaphragm thickness, mobility, and thickening fraction were measured by ultrasound.

**Results:**

The right and left diaphragm thickness, mobility, thickness variation, thickening fraction, and mobility were lower in individuals with COPD than in healthy individuals (p < 0.05). The left Functional Residual Capacity (FRC) diaphragm thickness, right Total Lung Capacity (TLC), and FRC diaphragm thickness were higher in mild COPD than moderate COPD and moderate COPD than severe COPD (p < 0.05). The right diaphragmatic thickening fraction and rate were higher in mild COPD than in moderate and severe COPD (p < 0.05). The left mobility was lower in severe COPD than in mild COPD (p < 0.05).

**Conclusion:**

Diaphragm ultrasound parameters decrease as disease severity increases in individuals with COPD. We think that adding diaphragm ultrasound parameters together with pulmonary function test to the evaluation of individuals with COPD will provide additional contributions to determining the course of the disease.

## 1. Introduction

Chronic Obstructive Pulmonary Disease (COPD) is a common, treatable, and preventable disease characterized by persistent respiratory signs and airway limitation due to airway and/or alveolar abnormality resulting from exposure to harmful gases or particles and causing abnormal lung development [1]. COPD is the leading cause of mortality and morbidity worldwide, causing significant social and economic burdens [2–4]. The World Health Organization (WHO) stated that COPD is the 3rd leading cause of death, and COPD-related deaths will be more than 4.5 million in 2030 [5,6]. While respiratory diseases constitute approximately 6% of total health expenditures in European Union countries, COPD constitutes 56% of these expenditures [7]. In individuals with COPD, chronic inflammation causes structural changes, narrowing of the small airways, and destruction of the lung parenchyma, resulting in loss of alveoli and decreased lung elastic recoil strength. Loss of small airways also causes airway limitation and mucociliary dysfunction that are characteristic of the disease [1,8].

In addition to pulmonary changes, extrapulmonary changes occur in individuals with COPD. Skeletal muscle dysfunction is among the most common extrapulmonary changes. Skeletal muscle dysfunction affects both respiratory and extremity muscles [9]. Respiratory muscle function is primarily characterized by respiratory muscle strength and endurance. Loss of strength and/or endurance causes diaphragm weakness and impaired performance [10]. In individuals with COPD, the diaphragm shortens due to pulmonary hyperinflation, the diaphragm is positioned in a nonoptimal position and works with a mechanical disadvantage, leading to respiratory muscle dysfunction [11–13]. It is stated that skeletal muscle thickness measurement is used in the evaluation of muscle loss in COPD, and diaphragm mobility is useful in the treatment and management of COPD. In the literature, there are studies examining diaphragm thickness, mobility, and thickening fraction in COPD and healthy individuals [14,15]. However, the number of studies examining the change of diaphragm thickness, mobility, and thickening fraction with disease severity in individuals with COPD is insufficient in the literature. Moreover, while diaphragm ultrasound parameters were evaluated separately in other studies in the literature; in this study, all of the diaphragm ultrasound parameters were evaluated. In addition, while these parameters were examined unilaterally in the literature; in this study, the right and left sides were examined separately. The aim of this study is to compare diaphragm thickness, mobility, and thickening fraction in individuals with COPD of different severity and healthy individuals and examine the relationship between these parameters and pulmonary function test parameters.

## 2. Materials and methods

A cross-sectional observational study design was used. The number of individuals to be included in the study was determined using the G*Power 3.1 software. It was observed that the effect size of the right diaphragm thickening fraction % results obtained in the reference study was at a strong level (d = 1.047). As a result of the sample size analysis carried out considering that a lower level of effect size (d = 0.9) could be obtained, it was calculated that 80% power could be obtained at a 95% confidence interval when at least 42 individuals (at least 21 for each group) were included in the study [14]. In the study, 59 male individuals (30 individuals with COPD and 29 healthy individuals) followed up by Bolu Abant İzzet Baysal University, Faculty of Medicine, Department of Chest Diseases were evaluated. The individuals included in the study were evaluated between October 2020/May 2021. According to the effect size of the difference between the groups (d = 1.325) in the % results of the right diaphragm thickening fraction obtained in these individuals, it was calculated that our study reached a power of 98.1% at a 95% confidence level.

Volunteers who were aged between 40–75 years and diagnosed with COPD and who did not change their medication for at least three weeks were included in the study. Individuals who had orthopedic, neurological, lung diseases other than COPD, unstable angina, a history of previous myocardial infarction, severe heart failure resistant to medical treatment, uncontrolled hypertension and cancer, who had undergone major surgery in the last six months and were in the exacerbation period of COPD were excluded from the study. Individuals without any diagnosed disease were included in the healthy group. The study was initiated after obtaining the necessary permission from Bolu Abant İzzet Baysal University (BAİBU) Clinical Research Ethics Committee (date: 19.10.2020 number: 420). Individuals were informed about the study, and a written consent form was acquired.

The individuals’ demographic information (name-surname, sex, age), height in meters (m), and body weight in kilograms (kg) were recorded. Furthermore, the body mass index (BMI) was recorded as kg/m^2^, using the ratio of body weight to height squared formula.

### 2.1. Pulmonary function test

Pulmonary functions were evaluated using a spirometer (Cosmed Microquark-PC Based Spirometer, Rome, Italy) [16]. Forced expiratory volume in the first second (FEV_1_), forced vital capacity (FVC), FEV_1_/FVC, peak expiratory flow (PEF), flow rate between 25% and 75% of vital capacity during forced expiration (FEF_%25–75_) and maximal expiratory flow (MEF 25%, 50%, 75%) were evaluated by the pulmonary function test [17]. Evaluations were repeated at least three times. The best of correct maneuvers was expressed as a percentage of expected values [18]. Individuals with COPD were classified according to the severity of airway limitation as mild (FEV_1_ ≥ 80%), moderate (50% ≤ FEV_1_ < 80%), severe (30% ≤ FEV_1_ < 50%), and very severe (FEV_1_ < 30%) [1].

### 2.2. Diaphragm thickness and mobility

**Figure 1:**
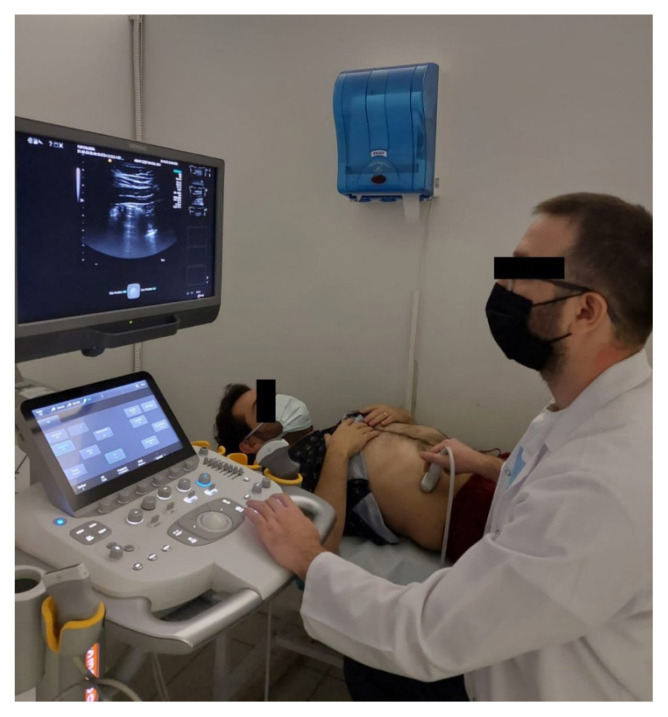
Diaphragm thickness and mobility measurement.

**Figure 2:**
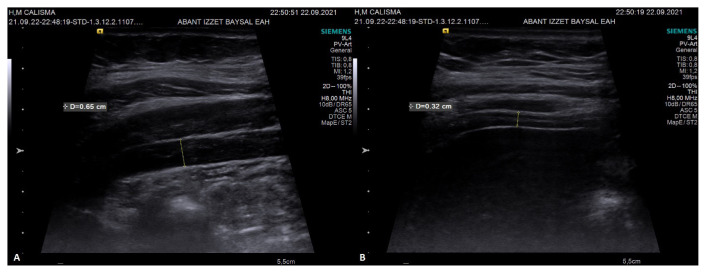
Diaphragm thickness measurement; A) TLC B) FRC.

**Figure 3:**
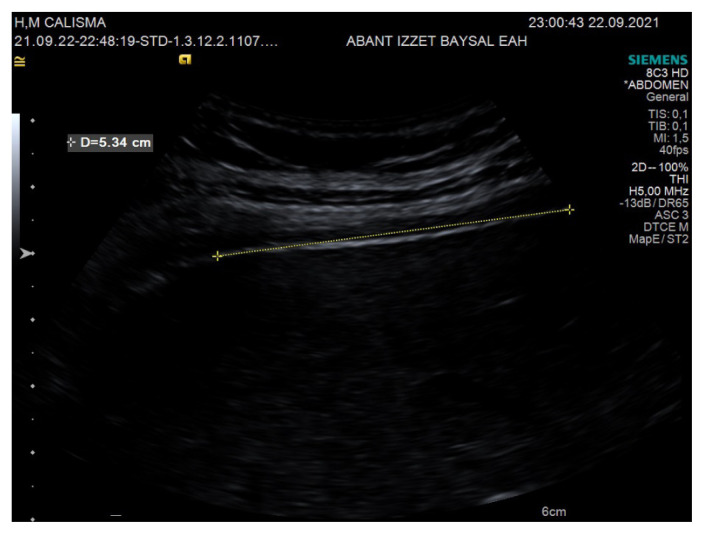
Diaphragm mobility measurement.

Diaphragm thickness and mobility were evaluated with the HI VISION Preirus ultrasound device (Hitachi Medical Systems, Tokyo, Japan). A 6–13 MHz linear probe was utilized for diaphragm thickness, whereas a 1–5 MHz convex probe was used for diaphragm mobility (Figure 1). Diaphragm thickness in individuals was measured twice, at the end of expiration for Functional Residual Capacity (FRC) and at the end of maximal inspiration for Total Lung Capacity (TLC). Diaphragm mobility was calculated as the displacement length of the apex of the diaphragm between the FRC and TLC lung volumes. Diaphragm thickness was measured from the 8^th^ or 9^th^ intercostal space, while diaphragm mobility was measured from the costal line junction of the medial axillary line (Figures 2,3). Diaphragm thickness and mobility measurements were repeated on the right and left sides [15,19]. The difference between the TLC and FRC diaphragm thicknesses was expressed as the diaphragm thickening, the ratio of the diaphragm thickening amount to the FRC diaphragm thickness was expressed as the diaphragm thickening fraction, and the ratio of the TLC diaphragm thickness to the FRC diaphragm thickness was expressed as the thickening ratio.

### 2.3. COPD assessment test

The COPD assessment test (CAT) was used in the evaluation of health status impairment in individuals with COPD. CAT is an 8-item test consisting of questions that evaluate the severity of symptoms such as shortness of breath, cough and sputum, and the impact of the disease on daily life. Each question is scored between 0–5 and the total score ranges from 0 to 40 points. A score of 0 represents the best and a score of 40 represents the worst state of health [20].

### 2.4. Charlson comorbidity index

Charlson comorbidity index was used to evaluate comorbidities. Charlson et al. in this index, comorbidities were scored between 1 and 6 according to disease severity. Individuals’ Charlson score was found by summing the scores determined for comorbidities [21].

### 2.5. MMRC dyspnea scale

The perception of shortness of breath during activities of daily living was evaluated using the MMRC Dyspnea Scale. Individuals with COPD were asked to choose the statement that best described the severity of dyspnea among 5 statements scored between 0 and 4 [22].

### 2.6. Statistical analysis

Data were analyzed using SPSS 25.0 (IBM SPSS Statistics 25 software (Armonk, NY: IBM Corp.)) packaged software. Continuous variables were expressed as mean ± standard deviation, median (minimum–maximum values), and categorical variables were expressed as numbers and percentages. The conformity of the data to the normal distribution was examined using the Shapiro-Wilk test. In the analysis of independent group differences, the independent samples t-test and One Way Analysis of Variance (post hoc: Tukey Test) were used when parametric test assumptions were provided, and the Mann-Whitney U test and Kruskal Wallis Variance Analysis (post hoc: Mann Whitney U test with Bonferroni correction) were used when assumptions were not provided. Chi-square analysis was performed to analyze differences between categorical variables. Moreover, Spearman’s correlation analysis was conducted to examine the relationships between continuous variables. The value of p < 0.05 was considered statistically significant in all analyses.

## 3. Results

**Figure 4:**
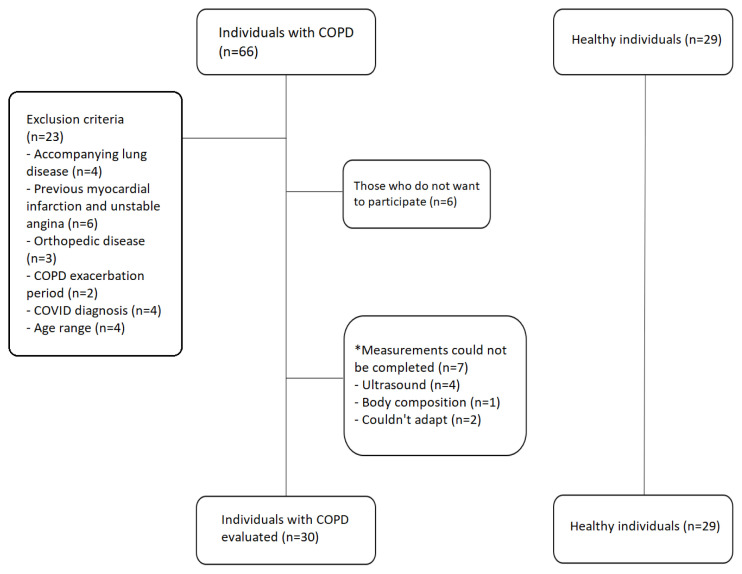
Participants flow chart.

The flow chart of the participants is shown in Figure 4. Upon comparing the age, height, body weight, and BMI values of individuals, there was no significant difference between individuals with COPD and healthy individuals (p > 0.05). There was a significant difference between individuals with COPD and healthy individuals in terms of smoking history, FEV1, FVC, FEV1/FVC, PEF, and FEF25–75% (p < 0.05) (Table 1).

When the diaphragm ultrasound parameters of individuals with COPD and healthy individuals were compared, all parameters were statistically decreased in individuals with COPD compared to healthy individuals (p < 0.05) (Table 2).

The comparison of diaphragm ultrasound parameters in individuals with COPD of different severity and healthy individuals is shown in Table 3.

FEV_1_%, FVC%, PEF%, MEF 25%, MEF 50%, and MEF 75% were positively correlated with the left and right TLC diaphragm thickness, FRC diaphragm thickness, mobility, and right diaphragm thickness variation (p < 0.05). FEV_1_%, FVC%, PEF%, MEF 25%, MEF 50%, and MEF 75% were positively correlated with the left diaphragm thickness variation (p < 0.05). FEV_1_%, FVC%, and MEF 50% were positively correlated with the right diaphragm thickening fraction % and thickening ratio (p < 0.05). The left diaphragm thickening fraction % and left diaphragm thickening ratio values were not correlated with any pulmonary function test parameters (p > 0.05). Diaphragm ultrasound parameters were not correlated with BMI, CAT, and CCI score (p > 0.05). Left and right TLC diaphragm thickness, FRC diaphragm thickness mobility were negatively correlated with MMRC dyspnea score (p < 0.05); left and right diaphragm thickening, diaphragm thickening fraction and ratio were not correlated with MMRC dyspnea score (p > 0.05) (Table 4).

## 4. Discussion

In this study, it was observed that diaphragm thickness, mobility, and thickening fraction decreased as COPD severity increased, and these parameters were associated with pulmonary function test parameters. Abd El Aziz et al. found that diaphragm thickness decreased at TLC, FRC, and RV in individuals with COPD, while Okura et al. revealed that TLC diaphragm thickness decreased in individuals with COPD compared to healthy individuals, and there was no difference in FRC and RV thicknesses between the three groups in their study conducted with 38 individuals with COPD, and 15 young and 15 elderly healthy male individuals [23,24]. Jain et al. determined that TLC and FRC diaphragm thickness decreased in individuals with mild and moderate COPD, and FRC diaphragm thickness increased in individuals with severe COPD [25]. While the reason for the increase in diaphragm thickness could not be fully explained, it was stated that it might be due to the development of some adaptations such as collagen deposition in severe obstruction. Ogan et al. stated that the maximum (deep inspiration) and minimum (tidal volume) diaphragm thicknesses were normal in individuals with COPD due to the adaptation of the diaphragm because of excessive work against increased mechanical load [26]. Elsawy revealed that TLC and RV diaphragm thicknesses were preserved in individuals with COPD due to the sarcomere adaptation of the muscle fiber protecting the static thickness of the diaphragm and compensatory overuse hypertrophy with the increase in COPD severity and hyperinflation [14]. In our study, TLC and FRC diaphragm thicknesses were found to be decreased in individuals with COPD compared to healthy individuals. Based on the studies in the literature, we think that this may be due to the etiological factors (such as systemic inflammation, oxidative stress, and drugs) that cause respiratory muscle dysfunction, especially mechanical changes due to hyperinflation.

Paulin et al. showed that diaphragm mobility decreased in individuals with COPD [27]. They stated that the main reason for the decrease in diaphragm mobility was air trapping and it was not affected by pulmonary hyperinflation. Yamaguti et al. also demonstrated that decreased diaphragm mobility in individuals with COPD was associated with air trapping rather than pulmonary hyperinflation and respiratory muscle strength [28]. In this study, they explained that abnormal diaphragm mobility reflecting the respiratory muscle dysfunction was mainly caused by the abnormal flow-volume performance of the lung in individuals with COPD. Similar to these studies, Shiraishi et al. also showed that diaphragm mobility decreased in individuals with COPD in comparison with the control group [29]. Corbellini et al. on the other hand found that diaphragm mobility in deep inspiration decreased in individuals with COPD due to static pulmonary air trapping and dynamic pulmonary hyperinflation compared to healthy individuals, but diaphragm mobility was higher during resting breathing in individuals with COPD due to the increase in inspiratory effort caused by pulmonary hyperinflation [15]. Jain et al. revealed that diaphragm mobility decreased in individuals with mild COPD compared to the control group, and it increased in individuals with moderate and severe COPD [25]. The researchers stated that the decrease in diaphragm mobility was due to air trapping and hyperinflation, and they could not fully explain the reason for the increase in diaphragm mobility. However, it was indicated that increased airway obstruction might lead to hypoxia and hyperventilation resulting in increased diaphragm mobility. In our study, it was observed that diaphragm mobility decreased in individuals with COPD. When other studies in the literature are reviewed, we think that this may be due to the shortening of the apposition region because of the lack of piston-like movement of the diaphragm as a result of air trapping in individuals with COPD and the structural changes of the diaphragm.

The diaphragm thickening fraction is used as an indirect measure of muscle fiber contraction, similar to the ejection fraction of the heart. It has recently been shown that the diaphragm thickening fraction is more sensitive than diaphragm thickness measurement in reflecting diaphragm contraction [30]. Elsawy found no difference in diaphragm thickness between the COPD and control groups but revealed that the diaphragm thickening fraction was lower in individuals with COPD [14]. According to this result, it was revealed that the contractility of the diaphragm might change in individuals with COPD. Abd El Aziz et al. also found that the amount of diaphragm thickening decreased in individuals with COPD [23]. Baria et al. on the other hand stated that the thickening ratio and diaphragm thickness did not change in individuals with COPD and that diaphragm dysfunction might reflect the mechanical impairment of diaphragm mobility secondary to pulmonary hyperinflation rather than physiological changes in contractility [19]. In our study, it was observed that the thickening fraction, the amount of thickening, and the thickening ratio decreased in individuals with COPD. According to our inferences from other studies in the literature, we think that the reason for this may be the force-length relationship that limits the contractility as a result of the shortening of the diaphragm, which is positioned in a nonoptimal position due to hyperinflation. In this study, it was determined that the right side diaphragmatic thickening fraction and ratio changed according to the severity of the disease; on the left side, it was seen that it did not change. We think that this is because the left hemidiaphragm has a smaller range of motion due to the spleen and its movement is restricted by the enlarged lung. Amin et al. also stated that the right side mobility is higher than the left side in diaphragm mobility due to the anatomical structure originating from the spleen [31]. Moreover; the right diaphragm displacement may be more pronounced because the dome is more prominent in the right hemidiaphragm due to the weight of the heart. Decreased left diaphragmatic mobility may affect the thickening fraction and ratio.

Abd El Aziz et al. demonstrated that diaphragm thicknesses at TLC, FRC, and RV decreased with the increase in COPD severity and were associated with FEV_1_, FVC, and FEV_1_/FVC [23]. El-hay et al. also stated that TLC and FRC diaphragm thicknesses decreased with disease severity [32]. It was indicated in this study that the decrease in diaphragm thickness in individuals with severe COPD might be due to a high FRC/TLC ratio in addition to the weakened diaphragm. Ogan et al. found no relationship between diaphragm thickness and FEV_1_ [26]. In their study, they emphasized that pulmonary hyperinflation affected diaphragm mobility rather than diaphragm thickness, and mobility better-reflected diaphragm function. Smargiassi et al. showed that TLC diaphragm thickness was associated with air trapping parameters (directly with IC/TLC, inversely with FRC/TLC and RV/TLC) [33]. In conclusion, they suggested that TLC diaphragm thickness could be a useful tool to predict pulmonary hyperinflation. Elsawy showed that the thickening fraction decreased as the severity of the disease increased [14]. However, FEV_1_% and FEV_1_/FVC were associated with thickening fraction, whereas FVC% and FEF_25–75%_ were not. In this study, it was observed that the most important factors affecting the thickening fraction were FEV_1_%, FVC%, and FEF_25–75%_. These results demonstrated that the diaphragm suffers from mechanical disadvantages (nonoptimal length-tension relationship of muscle fibers) with increasing COPD severity, and thus contractility decreases. Smargiassi et al. showed that the amount of thickening was associated with hyperinflation, air trapping (directly with IC/TLC, inversely with FRC/TLC and RV/TLC), and dynamic pulmonary volumes (VC, FRC, and FEV_1_) [33]. Therefore, airflow restriction resulting in air trapping and pulmonary hyperinflation was demonstrated to play a major role in the dynamic thickening process. Likewise, Hafez et al. based the association of thickening fraction with FEV_1_ on the fact that airway obstruction, which increases with the progression of COPD, causes dynamic air trapping and limits its contractility [34]. Corbellini et al. demonstrated that diaphragm mobility was associated with IC and IC/TLC in their study examining individuals with moderate, severe, and very severe COPD and stated that this was caused by the effect of dynamic pulmonary hyperinflation on diaphragm mobility [15]. Yamaguti et al. also showed that diaphragm mobility was strongly associated with FEV_1_ and parameters reflecting air trapping (RV and RV/TLC) and weakly associated with pulmonary hyperinflation (TLC) [28]. In the study, it was concluded that there was a close interaction between impaired respiratory mechanics and the severity of abnormal pulmonary function in COPD patients. In our study, in parallel to the literature, PFT parameters other than FEV_1_/FVC were found to be associated with diaphragm thickness, diaphragm mobility, and amount of diaphragm thickening. We think that the reason for this is mechanical changes that occur with air trapping and pulmonary hyperinflation resulting from the increase in disease severity. There is a need for new studies explaining the reasons for the relationship between diaphragm ultrasound parameters and PFT parameters. Moreover, the thickening fraction and ratio on the right side were associated with FEV1%, FVC%, and MEF 50%; no association was found on the left side. We think that this is due to the reduced range of motion in the left hemidiaphragm due to anatomical reasons. Diaphragm ultrasound parameters were not associated with BMI, symptom, and comorbidity score; however, it was associated with dyspnea. We think that this may be due to the fact that the deterioration in respiratory mechanics due to the decrease in diaphragm movement can increase the perception of dyspnea. Cimsit et al. found no correlation between diaphragm thickness and symptom scores (CAT and MMRC) [35]. Eryuksel et al. found no correlation between diaphragm thickening fraction and MMRC and CAT scores [36]. Rocha et al. found a relationship between diaphragm mobility and dyspnea perception. In the study, it was shown that changes in the diaphragm position make ventilation difficult, reduce respiratory capacity and increase the perception of dyspnea [37]. New studies are needed to explain the reasons for the relationship between diaphragm ultrasound parameters, PFT parameters, symptoms and comorbidity. Our study had limitation. Due to the high prevalence of COPD in males and patient flow, all of the individuals included in the study were male. Comparisons could be made in terms of gender by including female individuals in the assessment.

In conclusion, it was found that diaphragm thickness, mobility, and diaphragm thickening fraction decreased with the increase in disease severity in individuals with COPD, and diaphragm ultrasound parameters were associated with pulmonary function test parameters. We think that adding diaphragm ultrasound parameters together with pulmonary function test to the evaluation of individuals with COPD will provide additional contributions to determining the course of the disease. Moreover, it would be useful to evaluate diaphragm ultrasound parameters in individuals with COPD whose clinical condition is unpredictable. It was thought that adding diaphragm ultrasound parameters in addition to a routine pulmonary evaluation in individuals with COPD would be clinically important. Moreover, there is a need for new studies examining individual-specific pulmonary rehabilitation programs to increase diaphragm thickness and mobility.

## Figures and Tables

**Table 1:** Demographic and clinical characteristics of individuals with COPD and healthy individuals, mean ± S.D., median (min–max) or n (%).

		COPD (n = 30)	Healthy (n = 29)	p
**Age (years)**		63 (40–73)	54 (47–72)	0.068
**Height (cm)**		167.13 ± 6.53	169.34 ± 6.64	0.202
**Weight (kg)**		75.61 ± 16.17	81.83 ± 10.46	0.086
**BMI (kg/m** **^2^** **)**		27.05 (17.3–39.5)	27.8 (23.5–39.1)	0.228
**Smoking (pack** * **year)**		46.5 (0–85)	6 (0–18)	0.0001*
**FEV** **_1_**		70.13 ± 20.09	96.28 ± 16.29	0.0001*
**FVC**		89 (36–115)	100 (76–143)	0.007*
**FEV** **_1_** **/FVC**		72.05 (40.7–80)	79.3 (70.3–88.7)	0.0001*
**PEF**		63.5 ± 20.78	78.52 ± 20.24	0.007*
**FEF** **_25_**_–_ **_75%_**		42.77 ± 21.63	80.14 ± 25.11	0.0001*
**CAT**		12.47 ± 7.19	11 (4–29)	-
**CCI**		2 (1–9)	0 (0–2)	0.0001*
**Paroxysmal nocturnal dyspnea**	**Yes**	12 (40)	0 (0)	0.001*
	**No**	18 (60)	29 (100)	
**Orthopnea**	**Yes**	13 (43.33)	0 (0)	0.001*
	**No**	17 (56.67)	29 (100)	
**Resting shortness of breath**	**Yes**	22 (73.33)	0 (0)	0.001*
	**No**	8 (26.67)	29 (100)	
**Activity shortness of breath**	**Yes**	30 (100)	26 (89.66)	0.112 γ
	**No**	0 (0)	3 (10.34)	
**MMRC**	**Grade 0**	1 (3.33)	22 (75.86)	0.0001*
	**Grade 1**	6 (20)	7 (24.14)	
	**Grade 2**	5 (16.67)	0 (0)	
	**Grade 3**	12 (40)	0 (0)	
	**Grade 4**	6 (20)	0 (0)	

* significant p value less than 0.05; t: independent samples t-test; z: Mann-Whitney U test; χ^2^: Chi-square test;

γ Fisher exact chi-square test;

BMI; Body mass index; FEV_1:_ Forced expiratory volume in the first s; FVC: Forced vital capacity; PEF: Peak expiratory flow; FEF_%25–75:_ Flow rate between 25% and 75% of vital capacity during forced expiration; CAT: COPD assessment test; CCI: Charlson comorbidity index; MMRC: Modified Medical Research Council dyspnea scale.

**Table 2:** Comparison of the diaphragm ultrasound parameters values in individuals with COPD and healthy individuals, mean ± S.D., median (min–max).

	COPD (n = 30)	Healthy (n = 29)	p
**Left TLC diaphragm thickness (mm)**	4.13 ± 1.24	7 ± 0.33	0.0001*
**Left FRC diaphragm thickness (mm)**	2.88 ± 0.76	3.73 ± 0.22	0.0001*
**Left diaphragm thickening (mm)**	1 (0.5–2.2)	3.3 (2.8–3.6)	0.0001*
**Left diaphragm thickening fraction %**	44.15 ± 11.78	87.53 ± 4.68	0.0001*
**Left diaphragm thickening ratio**	1.46 (1.13–1.68)	1.86 (1.81–1.97)	0.0001*
**Left mobility (mm)**	44.4 (28.1–51)	58.2 (48.7–66.8)	0.0001*
**Right TLC diaphragm thickness (mm)**	4.27 ± 1.27	7.08 ± 0.26	0.0001*
**Right FRC diaphragm thickness (mm)**	3 ± 0.79	3.78 ± 0.21	0.0001*
**Right diaphragm thickening (mm)**	1 (0.5–2.3)	3.3 (3.1–3.5)	0.0001*
**Right diaphragm thickening fraction %**	41.74 ± 10.06	87.53 ± 4.78	0.0001*
**Right diaphragm thickening ratio**	1.41 ± 0.1	1.87 ± 0.05	0.0001*
**Right mobility (mm)**	44.9 (27.6–52)	59.8 (48.8–65.9)	0.0001*

* significant p value less than 0.05;

SD: Standard deviation; Min: Minimum; Max: Maximum; t: independent samples t-test; z: Mann-Whitney U test; TLC: Total lung capacity; FRC: Functional residual capacity.

**Table 3:** Comparison of diaphragm ultrasound parameters values in individuals with COPD of different severity mean ± S.D., median (min–max).

	Mild (1)	Moderate (2)	Severe (3)	p
	(n = 11)	(n = 13)	(n = 6)	
**Left TLC diaphragm thickness (mm)**	5.4 (3.7–6.3)	3.4 (3–6.1)	2.95 (1.7–3.1)	0.0001* (Kwh = 19,161) (1–2, 1–3, 2–3)
**Left FRC diaphragm thickness (mm)**	3.51 ± 0.43	2.8 ± 0.64	1.92 ± 0.17	0.0001* (F = 19,811) (1–2, 1–3, 2–3)
**Left diaphragm thickening (mm)**	1.9 (0.9–2.2)	1 (0.5–1.9)	1 (0.7–1.1)	0.003* (Kwh = 11,715) (1–2,1–3)
**Left diaphragm thickening fraction %**	52.5 (32.14–62.85)	39.13 (13.88– 47.61)	50 (36.84– 68.75)	0.003* (Kwh = 11,862) (1–2)
**Left diaphragm thickening ratio**	1.52 (1.32–1.62)	1.39 (1.13–1.47)	1.5 (1.36–1.68)	0.003* (Kwh = 11,729) (1–2)
**Left mobility (mm)**	48.5 (46.6–51)	43.6 (34–47.6)	33.2 (28.1–34)	0.0001* (Kwh = 23,687) (1–2,1–3)
**Right TLC diaphragm thickness (mm)**	5.45 ± 0.78	4 ± 0.91	2.72 ± 0.28	0.0001* (F = 25,092) (1–2, 1–3, 2–3)
**Right FRC diaphragm thickness (mm)**	3.62 ± 0.44	2.96 ± 0.65	1.93 ± 0.15	0.0001* (F = 21,008) (1–2, 1–3, 2–3)
**Right diaphragm thickening (mm)**	2 (1–2.3)	0.9 (0.7–1.9)	0.85 (0.5–0.9)	0.0001* (Kwh = 18,113) (1–2,1–3)
**Right diaphragm thickening fraction %**	50.21 ± 8.91	35.2 ± 6.57	40.38 ± 7.34	0.0001* (F = 11,583) (1–2, 1–3)
**Right diaphragm thickening ratio**	1.5 ± 0.09	1.35 ± 0.07	1.4 ± 0.07	0.0001* (F = 11,556) (1–2, 1–3)
**Right mobility (mm)**	49 (47.8–52)	43.5 (35.1–49.7)	34.5 (27.6–35.6)	0.0001* (Kwh = 22,430) (1–2,1–3)

* significant p value less than 0.05; F: One-Way analysis of variance (post hoc: Tukey Test); Kwh: Kruskal Wallis H test (post hoc: Mann Whitney U test with Bonferroni correction).

**Table 4:** The relationship between diaphragm ultrasound parameters and pulmonary function test parameters, BMI, MMRC, CAT, CCI score in individuals with COPD.

		FEV_1_%	FVC%	FEV_1_/FVC	PEF%	FEF %25–75	MEF %25	MEF %50	MEF %75	BMI	MMRC	CAT	CCI

**Left TLC diaphragm thickness (mm)**	**r**	0.906*	0.768*	0.222	0.535*	0.728*	0.816*	0.774*	0.808*	−0.003	−0.468*	−0.270	0.077
	**p**	<0.001	<0.001	0.239	0.002	<0.001	<0.001	<0.001	<0.001	0.986	0.009	0.149	0.687

**Left FRC diaphragm thickness (mm)**	**r**	0.893*	0.727*	0.279	0.555*	0.745*	0.857*	0.771*	0.818*	0.060	−0.484*	−0.272	0.058
	**p**	<0.001	<0.001	0.135	0.001	<0.001	<0.001	<0.001	<0.001	0.753	0.007	0.145	0.763

**Left diaphragm thickening (mm)**	**r**	0.611*	0.522*	0.045	0.329	0.450*	0.463*	0.493*	0.460*	−0.185	−0.210	−0.062	0.142
	**p**	<0.001	0.003	0.814	0.076	0.013	0.010	0.006	0.011	0.327	0.265	0.746	0.454

**Left diaphragm thickening fraction %**	**r**	0.203	0.100	−0.038	0.010	0.125	0.135	0.237	0.055	−0.209	0.271	0.246	0.181
	**p**	0.283	0.598	0.843	0.956	0.510	0.477	0.207	0.774	0.269	0.148	0.189	0.339

**Left diaphragm thickening ratio**	**r**	0.204	0.101	−0.039	0.015	0.126	0.137	0.234	0.053	−0.205	0.273	0.243	0.178
	**p**	0.280	0.597	0.839	0.938	0.507	0.472	0.213	0.779	0.276	0.144	0.196	0.347

**Left mobility (mm)**	**r**	0.979*	0.818*	0.264	0.571*	0.760*	0.833*	0.861*	0.883*	−0.100	−0.430*	−0.261	0.048
	**p**	<0.001	<0.001	0.159	0.001	<0.001	<0.001	<0.001	<0.001	0.599	0.018	0.164	0.801

**Right TLC diaphragm thickness (mm)**	**r**	0.907*	0.746*	0.221	0.547*	0.738*	0.787*	0.782*	0.819*	−0.017	−0.444*	−0.256	0.097
	**p**	<0.001	<0.001	0.241	0.002	<0.001	<0.001	<0.001	<0.001	0.931	0.014	0.171	0.609

**Right FRC diaphragm thickness (mm)**	**r**	0.898*	0.732*	0.245	0.544*	0.751*	0.835*	0.767*	0.811*	0.039	−0.506*	−0.279	0.068
	**p**	<0.001	<0.001	0.192	0.002	<0.001	<0.001	<0.001	<0.001	0.839	0.004	0.136	0.720

**Right diaphragm thickening (mm)**	**r**	0.792*	0.714*	0.081	0.376*	0.555*	0.559*	0.658*	0.658*	−0.154	−0.215	−0.090	0.029
	**p**	<0.001	<0.001	0.669	0.041	0.001	0.001	<0.001	<0.001	0.418	0.254	0.637	0.877

**Right diaphragm thickening fraction %**	**r**	0.453*	0.364*	−0.035	0.031	0.295	0.304	0.456	0.283	−0.154	0.212	0.205	−0.029
	**p**	0.012	0.048	0.856	0.871	0.113	0.102	0.011	0.129	0.417	0.261	0.278	0.877

**Right diaphragm thickening ratio**	**r**	0.447*	0.361*	−0.041	0.029	0.288	0.303	0.445*	0.276	−0.155	0.222	0.207	−0.028
	**p**	0.013	0.050	0.831	0.880	0.122	0.104	0.014	0.140	0.412	0.238	0.271	0.882

**Right mobility (mm)**	**r**	0.951*	0.781*	0.269	0.632*	0.760*	0.822*	0.837*	0.883*	−0.057	−0.485*	−0.342	0.142
	**p**	<0.001	<0.001	0.150	<0.001	<0.001	<0.001	<0.001	<0.001	0.763	0.007	0.064	0.455

* significant p value less than 0.05;

r: Spearman correlation coefficient; TLC: Total lung capacity; FRC: Functional residual capacity; FEV_1:_ Forced expiratory volume in the first s; FVC: Forced vital capacity; PEF: Peak expiratory flow; FEF_%25–75:_ Flow rate between 25% and 75% of vital capacity during forced expiration; MEF: Maximal expiratory flow; BMI: Body mass index; MMRC: Modified Medical Research Council dyspnea scale; CAT: COPD Assessment test; CCI: Charlson comorbidity index.
